# Mediated Non-geminate Recombination in Ternary Organic Solar Cells Through a Liquid Crystal Guest Donor

**DOI:** 10.3389/fchem.2020.00021

**Published:** 2020-02-11

**Authors:** Ao Yin, Dongyang Zhang, Jianqiu Wang, Huiqiong Zhou, Zhiqiang Fu, Yuan Zhang

**Affiliations:** ^1^School of Chemistry, Beijing Advanced Innovation Center for Biomedical Engineering, Beihang University, Beijing, China; ^2^CAS Key Laboratory of Nanosystem and Hierachical Fabrication CAS Center for Excellence in Nanoscience, National Center for Nanoscience and Technology, Beijing, China; ^3^School of Engineering and Technology, China University of Geosciences, Beijing, China

**Keywords:** ternary solar cells, charge recombination, charge transfer states, small molecule donor, voltage loss

## Abstract

The approach via ternary blends prompts the increase of absorbed photon density and resultant photocurrent enhancement in organic solar cells (OSCs). In contrast to actively reported high efficiency ternary OSCs, little is known about charge recombination properties and carrier loss mechanisms in these emerging devices. Here, through introducing a small molecule donor BTR as a guest component to the PCE-10:PC_71_BM binary system, we show that photocarrier losses via recombination are mitigated with respect the binary OSCs, owing to a reduced bimolecular recombination. The gain of the fill factor in ternary devices are reconciled by the change in equilibrium between charge exaction and recombination in the presence of BTR toward the former process. With these modifications, the power conversion efficiency in ternary solar cells receives a boost from 8.8 (PCE-10:PC_71_BM) to 10.88%. We further found that the voltage losses in the ternary cell are slightly suppressed, related to the rising charge transfer-state energy. These benefits brought by the third guest donor are important for attaining improvements on key photophysical processes governing the photovoltaic efficiencies in organic ternary solar cells.

## Introduction

The recent efforts on organic bulk heterojunction (BHJ-OSCs) solar cells have pushed forward this photovoltaic technology toward a meaningful solution for generating the electricity at lower expenses. An efficient strategy to further boost the photon-harvesting in BHJ-OSCs concerns ternary blends that are capable of capturing a larger portion of the solar spectrum. (Lu et al., 2014; Li et al., 2017). As a result, the power conversion efficiencies (PCE) have exceeded 10% (Kan et al., 2017) with PCEs > 14% using blends of two non-fullerene acceptors (Xiao et al., 2017). The general concept of ternary OSCs relies on addition of a third photo-absorber into the prime binary BHJ to achieve modulations on solar cell performance. A successful design for ternary OSCs involve introducing a small molecule (SM) donor into the polymer blends where the carrier transport profits from the high crystallinity of SM donors (Zhang et al., 2017a). Ternary blends with two co-blended SM-donors also have been reported showing enlarged PCEs (Baran et al., 2016). Recently, there have emerged ternary blends comprising of two well-miscible acceptors with which the photocurrent can increase due to the complementary absorption of acceptor alloy (Jiang et al., 2017b). Given the intrinsic trade-off between short-circuit current (*J*_sc_) and open-circuit voltage (*V*_oc_) in organic BHJ-OSCs, implementation of a concurrent increase in these two parameters is yet of challenge. To this end, maximizing the gain of photocurrent while maintaining voltage losses (Δ*V*_oc_) unchanged appears to be significant to reach the potential of ternary BHJ-OSCs.

In contrast to actively reported progressions in the PCE of ternary OSCs, fundamental insights into charge transport and recombination properties still lack for these devices. Although there has been a consensus on the general design rules for ternary BHJ-OSCs in terms of energetic level matching or morphology compatibility (Ye et al., 2012), questions including how the introduced third components impact charge recombination and carrier losses in ternary blends remain unclear. In previous studies based on fullerene binary OSCs, charge recombination has been identified as a major loss channel for the PCE (Julien et al., 2018). It is generally accepted that after interfacial exciton dissociation on charge transfer states (CTS) (Veldman et al., 2009), the fate of electron-hole (or polaron) pairs is mainly dictated by the competing processes of charge collection and recombination (Deibel et al., 2010). In addition to geminate losses at CTS, largely due to severe morphology reasons, non-geminate recombination tends to play a more critical role in ultimate photovoltaic characteristics (Hou et al., 2018). Non-geminate recombination often occurs via bimolecular paths (with insignificant charge trapping) which are subject to the encounter probability of the two carriers in the mutual coulombic field. In this scenario, the recombination rate (*B*) is given by (van der Poll Thomas et al., 2012).

(1) $$\begin{array}{l} B=\beta np, \end{array}$$

where β is the recombination rate constant (or coefficient), and *np* is product of mobile electron and hole densities under irradiation. In binary BHJ-OSCs, the β has been found to deviate from the Langevin rate β_L_, purely governed by the mobility of the two carriers as β_L_ = (*q*/ε_0_ε_r_)(μ_p_+μ_n_) (here *q* is elementary charge, ε_0_ε_r_ is the dielectric constant and μ_p_ or μ_n_ is the hole or electron mobility) (Proctor Christopher et al., 2014). The reduction of recombination in BHJs leads to a so-called reduction factor (γ) defined as γ = β/β_L_. Values of γ < 1 have been observed in binary OSCs with non-fullerene acceptors, which can be correlated to the transport balance in the BHJ film. In addition, the significance of recombination has been related to its impact on the device fill factor (FF). For example, the dependencies of *B* on irradiation intensity (Armin et al., 2016; Brus Viktor et al., 2016; Heiber et al., 2016) have been linked to the observed variation of photocurrent with applied bias, which basically describes the shape of photocurrent or FF in device. In rationally-designed ternary systems, the enlargement of photon densities related to the guest components should result in a boost of photocurrent. On the other hand, the increase of *n* (or *p*) tends to raise the encounter probability of photo-carriers, according to Equation 1. Therefore, attaining the control on β seems to be essential for efficient carrier sweepout such that the carriers can contribute to the photocurrents in ternary OSCs. In this context, it will be of interest to concern a guest molecular donor with a low recombination rate and examine how the recombination process in ternary blends is modulated in the presence of donor guest. So far, investigations in these aspects are rarely reported, which impedes further enhancement of PCEs in ternary OSCs.

**Figure 1:**
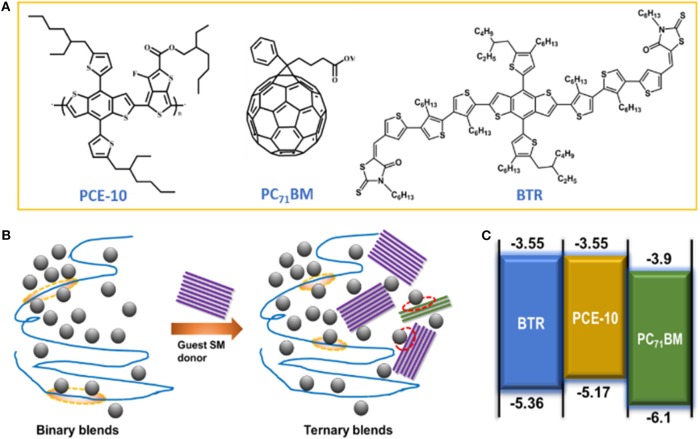
**(A)** Chemical structures of used components in studied ternary blends. **(B)** Illustration of binary blends comprising of a polymer donor and fullerene acceptor alongside ternary blends with addition of a third small molecule (SM) acceptor. Also highlighted by ovals are the interfaces between polymer donor/PC_71_BM (orange) and SM-donor/PC_71_BM (red). **(C)** Energy diagram of ternary blends in this study.

In this article, we chose a ternary model system comprising a polymeric donor ([4,8-bis(5-(2-ethylhexyl)thiophen-2-yl)-benzo[1,2-b:4,5-b′]dithiophene-co-3-fluorothieno[3,4-b]-thiophene-2-carboxylate]) PCE-10 (Liao et al., 2013) blended with PC_71_BM as the prime binary BHJ and a liquid crystal benzodithiophene terthiophene rhodanine (BTR) (Sun et al., 2015) as the third SM-donor guest (see chemical structure of different components in Figure 1A). We show that the low recombination rate in the BTR:PC_71_BM binary OSC provides an opportunity for mediating recombination losses in the PCE-10:BTR:PC_71_BM ternary devices. Based on an optimal blend ratio (0.2:0.8 w/w for BTR:PCE-10), the enlargements of *J*_sc_ and FF in the ternary cell are found associated with a stronger reduction factor with regard to the polymer binary device. As a result, the recombination rate constant and recombination intensity are both weakened. The modified recombination is further manifested by the recombination kinetics with light intensity (*P*_light_)-dependent impedance spectroscopy. We observe longer-lived photo-carriers in the ternary cell over a wide range of *P*_light_. The suppressed charge recombination in ternary devices leads to more efficient carrier extraction, which explains the simultaneously enhanced photocurrent and fill factor in the presence of BTR donor. Based on Fourier transform photocurrent spectroscopy, we gained insights into the voltage losses (Δ*V*_oc_) in the ternary OSCs. The results suggest a slightly reduced non-radiative recombination loss but unchanged radiative recombination loss due to the rising of charge transfer-state energy. This work enriches our fundamental understandings on charge recombination properties in ternary OSCs which coupled with smart device design may prompt the improvement of photovoltaic efficiencies.

## Experiment

### Materials

PCE-10, and BTR were purchased from 1-Material Inc., and PC_71_BM was purchased from Lumtec Inc. and used as received.

### Device Fabrication

Patterned indium-tin-oxide (ITO) glass substrates were cleaned sequentially in soap water and with socination using deionized water, acetone, and isopropanol. After drying with N_2_, the ITO substrates were UV/ozone treated for 4 min. ZnO solutions were prepared by dissolving 0.2 g of zinc acetate dihydrate [Zn(CH_3_COO)_2_·2H_2_O, 99.9%, Afla] and 0.055 ml of ethanolamine (NH_2_CH_2_CH_2_OH, 99.5%, Aladdin) in 2 mL of 2-methoxyethanol (CH_3_OCH_2_CH_2_OH, 99.8%, Alfa). ZnO films were prepared by spin-coating the precursor solution on top of the ITO substrates (5,000 rpm) for 20s as the electron transport layer followed by thermal annealing at 200°C for 30 min in air. The ZnO-coated ITO substrates were then transferred into a nitrogen-filled glovebox before use. PCE-10:PC_71_BM (1:1.1 weight ratio) and PCE-10:BTR:PC_71_BM (0.8:0.2:1.1 weight ratio) were solublized in chlorobenzene (15 mg/ml) with 2.5% DIO (v/v). The BTR:PC_71_BM (1:1.1 weight ratio) was dissolved in chloroform (15 mg/ml). The photoactive layers were attained by spin-coating the BHJ solutions pre-heated at 60°C over night at appropriate spin-rate, leading to typical film thickness of ~200 nm. After deposition of the BHJ films, drops of chloroform were dripped into a small dish holding the BTR devices with waiting for 40s. Then MoO_x_ was evaporated on the active layer as hole transport layer at a pressure of ~10^−5^ Pa. Finally, the Al cathode (~80 nm) was thermally evaporated on MoO_x_. The active area of the device was 4 mm^2^, defined by shadow masks. Single carrier device: the active layers were prepared identically to the procedure for solar cells. The hole-only devices were fabricated with the structure of ITO/PEDOT:PSS/active layer/Au and the structure for electron-only devices was ITO/ZnO/active layer/PFN-Br/Al.

### Characterization

*J-V* characteristics of solar cells was performed by using a Keithley 2400 Sourcemeter under AM 1.5G solar illumination at 100 mW/cm^2^ provided by a Class AAA solar simulator along with a National Institute of Metrology (NIM, China) calibrated KG5-filtered silicon reference cells. Irradiation-dependent solar cell testing was performed by applying a filter wheel with designed optical densities between the samples and light source to obtain desired illumination intensities and calibrated by a NIM-certified silicon reference cell. Single carrier devices were characterized in a Lakeshore vacuum probe station by using a Keithley 4,200 semiconductor parameter analyzer in dark condition. PL spectroscopy of BHJ and neat films was measured by using a Horiba Jobin Yvon Nanolog fluorimeter under excitation of 580 nm.

## Results and Discussion

**Figure 2:**
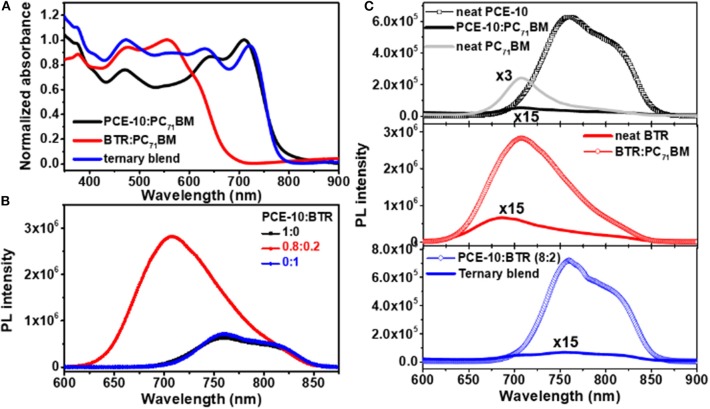
**(A)** Normalized absorption spectroscopy of binary and ternary blend films. **(B)** Photoluminescence (PL) quenching based on neat donor(s) and binary (ternary) blend films. **(C)** PL spectroscopy of neat and co-blended donor films.

Figure 1B schematically illustrates the nanomorphology of PCE-10:PC_71_BM binary and ternary blends with the co-blended BTR SM-donor. As can be seen, additional interfaces at BTR/fullerene are introduced in the ternary blends which likely create increased recombination channels. The chosen liquid crystal BTR donor possesses a deeper-lying energy of the highest occupied molecular orbital (HOMO) at ~5.36 eV and an absorption between 400 and 550 nm. This feature enables a complementary absorption to that of the PCE-10:PC_71_BM binary blends (see energy diagram of ternary blends in Figure 1C). Hereinafter, we focus on the analysis based on the optimal blend ratio at 0.8:0.2 (w/w) for PCE-10:BTR that yields the best PCE of 10.88%. (Chen et al., 2017) With non-optimal blend ratios (for PCE-10:BTR), more significant geminate losses may be present, which complicates our analyses on bimolecular recombination properties. Figure 2A displays absorption spectra of the binary and ternary blend films normalized to respective absorption peaks. Upon addition of BTR, the ternary blend film exhibits a broader absorbance with a spectral range between 400 and 750 nm. This optical profile is expected to promote *J*_sc_ in the solar cell, which will be detailed below.

In addition to the broadened absorption, it is a prerequisite that the singlet excitons created in the guest donor of ternary blends can dissociate into mobile carriers to contribute to the photocurrent (Lu et al., 2014). In this regard, we examined the photoluminescence (PL) quenching efficiency (PL_quench_) by which charge transfer in ternary BHJ films was assessed (Ameri et al., 2013). Figure 2B shows steady-state PL spectroscopy measured on thin films of neat donors, binary and ternary blends. As can be seen, the PCE-10:PC_71_BM binary and ternary bled films both display a strongly quenched PL at ~760 nm originating from singlet excitons in the PCE-10 donor (Chen et al., 2017), corresponding to a PL_quench_ of 99.88% (PCE-10 blends) and 99.39% (ternary blends). The quenching of PL from BTR (~708 nm) is also pronounced in the ternary blends, which is in contrast to the less significant quenching of the donor PL in BTR:PC_71_BM (Figure 2B) showing a PL_quench_ of 93%. This observation can be correlated to the reduced homogeneity in the SM binary blends, indicated by electron microscopy (Sun et al., 2011), pointing to a larger phase separation. To better assess charge transfers in the ternary blends, we also examined PL of co-blended donor films with the results shown in Figure 2C. With 20% of BTR, the PL of donor blends resembles that of neat PCE-10 and we cannot observe the emission from the SM donor (around 708 nm). The significant quenching of singlet excitons in BTR is suggestive of efficient hole transfers from the SM-donor toward PCE-10 polymer, which seems to be driven under the HOMO energy offset of ~200 meV (see Figure 1B). The PL results provides evidence for the occurrence of electron transfers at the donor/PC_71_BM and hole transfers at the BTR/PCE-10 interfaces in the ternary blends.

**Figure 3:**
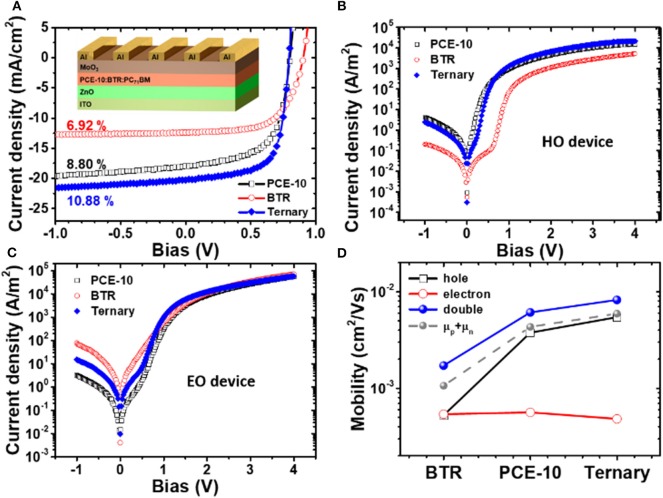
**(A)** Current density vs. voltage (*J-V*) characteristics of BHJ-OSCs based on photo-active layers of PCE-10:PC_71_BM, BTR:PC_71_BM binary blends, and PCE-10:BTR:PC_71_BM ternary blends. Dark *J-V* curves of **(B)** hole-only (HO) and **(C)** electron-only (EO) devices based on the same active layers of solar cells. **(D)** Carrier mobility of holes, electrons, and double carriers in BTR and PCE-10 binary, and ternary blends determined by single-carrier device measurements and solar cell dark current analysis. Also compared is the sum of electron and hole mobilities in single-carrier devices.

Now we turn to biomolecular recombination in ternary devices. As test beds, we fabricated ternary and corresponding binary BHJ-OSCs with an inverted device architecture (see device structure in the inset of Figure 3A). Figure 3A shows photocurrent density vs. voltage (*J-V*) characteristics of binary and ternary solar cells (with the optimal blend ratio of 0.8:0.2 for PCE-10:BTR) and the extracted device parameters are summarized in Table 1. The PCE-10 and BTR binary solar cells produce a PCE of 8.8 and 6.92%, respectively at the optimized conditions (the D/A ratios are both of 1:1.1 for cells with the polymer and SM-donors). Upon addition 20% of BTR guest donor into the polymer binary blend, the ultimate PCE receives a considerable enhancement to 10.88%, showing an enhanced *J*_sc_ = 20.34 mA/cm^2^ and FF = 69.02%. A slight increase in *V*_oc_ (22 meV) was found in the ternary device which can be ascribed to the deeper-lying HOMO in BTR. The *V*_oc_ result is in line with the general tendency in ternary solar cells where the *V*_oc_ takes advantage of the binary system having a larger effective band gap, defined by the energetic offset between donor HOMO and acceptor LUMO (Scharber et al., 2006).

**Table 1:** Photovoltaic parameters of PCE-10:PC_71_BM and BTR:PC_71_BM binary, and PCE-10:BTR:PC_71_BM ternary solar cells (blend ratio is 0.8:0.2:1) under 1.5 AM G solar irradiation (100 mW/cm^2^).

**BHJ**	***V*_**oc**_ (V)**	***J*_**sc**_ (mA/cm^**2**^)**	**FF (%)**	***η* (%)**
PCE-10:BTR:PC_71_BM	0.794	20.34	69.02	10.88
PCE-10:PC_71_BM	0.772	17.62	64.52	8.80
BTR:PC_71_BM	0.904	12.45	61.76	6.92

To have more mechanistic understandings on the modified device performance, we examined charge transport in steady-state by single-carrier device measurements. It is noteworthy that charge carrier mobilities in organic semiconductor devices can be determined either by transient, e.g., photo-CELIV (Yang et al., 2015) or steady-state opto-electrical methods (Nicolai et al., 2012). While the former techniques often require us to use thick films, the attained results may not truly reflect actual conditions in the solar cell with film thicknesses of active layer around 100 nm in some cases. Also, the dispersive transport in disorder systems tends to challenge reliable analyses on the transit time extracted from transient techniques, eventually complicating the mobility determination (Wetzelaer Gert-Jan et al., 2013). With these considerations, we simply adopted the well-established steady-state method with which the mobility in the space-charge limited current (SCLC) regime was assessed. Figures 3B,C show *J-V* characteristics in dark of hole- and electron-only devices based on active layers prepared identically to those in solar cells. The mobility of holes (μ_p_) and electrons (μ_n_) in blend films were determined through fitting the measurements to Mott-Gurney law. (Laquai et al., 2015) The results of μ_p_ and μ_n_ were averaged based on 10 devices in respective conditions and are shown in Figure 3D. As can be seen, the BTR has moderate carrier mobility with a balanced mobility ratio approaching 1. As will be addressed in follows, such transport feature can be connected to the low recombination rate in the BTR: PC_71_BM binary system with which the recombination in ternary devices are modulated.

Upon addition of the BTR donor, we observe opposite changes in the mobility of the two carriers, i.e., μ_p_ in the ternary blend increases with respect to μ_p_ in the two binary systems, while μ_n_ becomes slightly reduced. This result may be related to the distinct impacts of BTR on the charge transport networks involving the two carriers. Note that in our devices, the mobility imbalance (with the largest mobility ratio of 13.8) is not too severe and thus it may not cause the space-charge effect in photocurrents (Mihailetchi et al., 2005). This argument is supported by the absence of square-root dependent photocurrent on effective bias (see results in Figure S1) (Lenes et al., 2009). In a previous study based on the ITIC electron acceptor, we show that the imbalanced carrier mobility in BHJ solar cells does not necessarily cause an enlargement of recombination, on the contrary it beneficially leads to a stronger reduction for bimolecular recombination (Zhang et al., 2017b). The presence of the recombination factor can be understood where faster carriers in the BHJ have to wait for their slower counterparts to recombine at the donor/acceptor interface, effectively weakening the encounter probability, and recombination intensity (Wehenkel et al., 2012). With this knowledge, the most imbalanced mobility in the ternary blends is implicative of a smaller reduction factor γ which in turn can mitigate recombination losses. To determine γ, we extracted the effective mobility of double carriers (μ_sol_) based on the dark current of solar cells with Mott-Gurney law fittings. As shown in Figure 3D, the μ_sol_ of the ternary device is higher than both of μ_p_ and μ_n_ in the binary systems. In solar cells, μ_sol_ is subject to the co-existing processes of charge recombination and neutralization (Maurano et al., 2010). To this end, the μ_sol_ cannot be simply calculated by the sum of single carrier mobilities (μ_p_+μ_n_). A μ_sol_ = μ_p_+μ_n_ only validates for double-carrier devices where the hole and electron transport independently within their own (non-percolated) networks (Heeger Alan, 2013) and never can meet with each other, resembling the operation of two back-connected single-carrier diodes (Wetzelaer Gert-Jan et al., 2013). Apparently this situation does not apply to the BHJ-OSCs featured with phase-separated and inter-percolated transport networks. As will be shown, that the μ_sol_ exceeds the value of μ_p_+μ_n_ mainly stems from the recombination current in the solar cell and the difference between these mobility values provides a quantification for γ based on a simple analytical model reported previously (Wetzelaer Gert-Jan et al., 2013).

**Figure 4:**
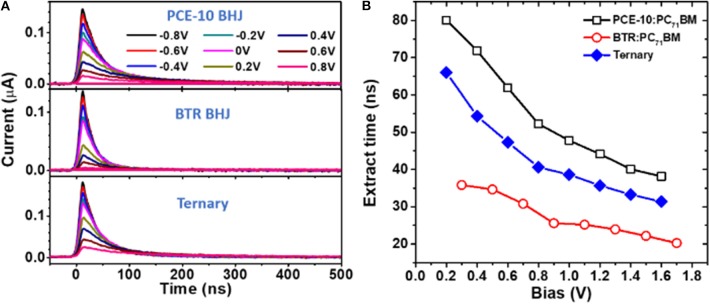
**(A)** Transient photocurrent (TPC) decay kinetics of different BHJ-OSCs measured with applying different biases (excitation at 488 nm). **(B)** Extraction time of charges in operating solar cells as a function of bias determined by fittings with a mono-exponential decay model.

To better correlate the steady-state transport to photovoltaic behaviors, we measured transient photocurrent (TPC) decay kinetics in the ternary and binary solar cells excited at 488 nm. The results of TPC at various biases are shown in Figure 4A. All the TPC trances are characteristics of a sharp rise in association with longer-lived decays extended to hundreds of nanoseconds. We note that the peak intensity reduces with incrementally varied forward bias, which results from the increased recombination current compensating the photocurrent at lower internal electrical fields (*E*_int_). With an invariable recombination in the solar cell, the bias-dependent decay traces in Figure 4A should overlap on top of each other after normalization. While the decay becomes evidently slower at larger forward bias (see normalized TPC in Figure S2), confirming that the recombination indeed varies at different *E*_int_.

Based on the mono-exponential decay model, we determined the extraction time for charges (τ_ext_) in the solar cells. Figure 4B shows τ_ext_ vs. bias characteristics for solar cells with ternary and binary blends. All devices exhibit a relatively fast charge sweepout with the τ_ext_ falling in dozens of ns. We note that the order of τ_ext_ does not exactly follow that of the carrier mobility or balance. For example, the τ_ext_ for the BRT binary cell amounts to the lowest values, followed by that of the ternary device and lastly the binary blend system. This non-correlation is understood by that the τ_ext_ is subject to the combined result of charge collection and recombination and both processes will be promoted with increased mobility. The fast carrier extraction in the SM-binary solar cell may be linked to the smaller recombination rate associated with a very balanced mobility. Also, the τ_ext_ in the BTR binary device displays the least field-dependence compared to PCE-10 based binary and ternary devices. The different dependencies of τ_ext_ on *E*_int_ seems not to arise from a field-dependent mobility, as the current in single-carrier devices exhibit nearly perfect quadratic voltage dependence within a wide bias range (see *J-V* characteristics in Figure S3). On the other hand, field-dependent charge generation has been observed in a variety of BHJ-OSCs which was used to explain the poor fill factor (Albrecht et al., 2012). While in our case, geminate losses at low electric fields tend not to be a significant factor given the relatively high FF. To this end, the different dependencies of τ_ext_ on *E*_int_ (or bias) could be related to the field dependence of non-geminate recombination in our BHJ devices (Credgington et al., 2012; Foertig et al., 2013). An important indication from the TPC measurements is that the introduced BTR donor promotes the efficiency for charge collection in the ternary solar cell in competition with recombination. As a result, the recombination losses with the increased density of photo-carriers can be suppressed.

The data so far, all point to reduced carrier losses in the ternary OSC with gains of *J*_sc_ and FF. To have a quantitative assessment on recombination, next we determined the recombination rate constant and reduction factor with the analytical model proposed by Wetzelaer Gert-Jan et al. (2013) which is written as,

(2) $$\begin{array}{l} \gamma =\frac{16\pi }{9}\frac{\mu_{p}\mu_{n}}{\mu_{sol}^{2}-{\left(\mu_{p}+\mu_{n}\right)}^{2}} \end{array}$$

**Figure 5:**
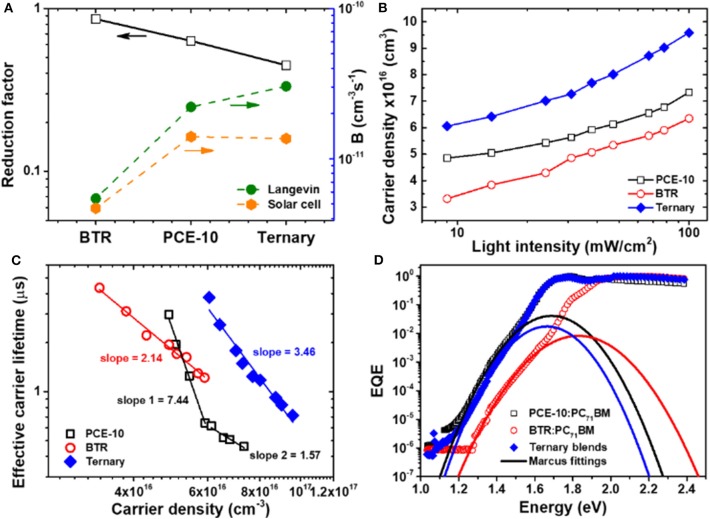
**(A)** Reduction factor γ, recombination rate constant β and Langevin rate constant β_L_ determined on BHJ-OSCs with PCE-10 and BTR binary blends and PCE-10:BTR:PC_71_BM ternary blends. **(B)** Density of photocarriers as a function of light intensity in binary and ternary devices. **(C)** Effective carrier lifetime vs. carrier density characteristics of solar cells alongside linear fittings used to assess the recombination order. Inset texts: slopes of linear fittings. **(D)** Fourier transform photocurrent spectra (FTPS) of binary and ternary OSCs together with fittings with Marcus equation.

The relevance of Equation 2 to solar cells is that the charge recombination parameters determined by transport measurements can mimic the situation in steady-state solar cell operation. Figure 5A shows the attained reduction factor γ for various solar cells. The γ displays a decreasing trend with the increase of mobility imbalance, e.g., the smallest value of 0.448 is found in the ternary with the largest mobility ratio. The correlation of γ to mobility ratio agrees with a recent study on PBDB-T:ITIC non-fullerene OSCs where the identified smaller γ is accompanied by a more severe mobility imbalance (Zhang et al., 2017b). A strong reduction in bimolecular recombination with respect to the Langevin process has been observed in non-fullerene binary OSCs with a large FF as well.

The Langevin rate constant β_L_ generally follows the relation,

(3) $$\begin{array}{l} \beta_{L}=\frac{q}{\varepsilon_{o}\varepsilon_{s}}\left(\mu_{p}+\mu_{n}\right) \end{array}$$

where ε_0_ε_s_ is the dielectric constant of the BHJ film. Based on Equation 3, we are able to extract the recombination rate constant in the solar cell according to β = γβ_L_. Figure 5A shows the determined β and the results are in line with typical values in BHJ-OSCs in the range of 10^−10^–10^−12^ cm^−3^s^−1^ (Jiang et al., 2017a). Of importance: the recombination rate constant in the ternary reduces when compared to the β in the prime binary blends. These results can benefit from the introduced BTR donor with the lowest value of *B* in blend with PC_71_BM. The modified bimolecular recombination in the ternary solar cell also is correlated to the π-π stacking in the ternary BHJ that was found to preferentially adapt to the out-of-plane direction with a small intermolecular *d*-space (~3.77 Å) (Zhang et al., 2015). The ternary BHJ with similar blend ratios have been identified with an improved domain purity with respect to that in the PCE-10:PC_71_BM film (Ran Niva et al., 2015). As bimolecular recombination in BHJ-OSCs primarily occurs at the D/A interface (Janssen and Jenny, 2013), the reduction of mixed phases with higher domain purity helps lower the encounter probability for photo-carriers. This tendency is fully consistent with the identified reduction in the recombination rate constant in our ternary device.

It should be noted that the determined recombination parameters in Figure 3A extracted from steady-state transport only provides an approximation on the average rate of recombination (Peter et al., 2014). In actual solar cells under irradiation, the β may not necessarily be a constant, but change with carrier density (*np*). Carrier density-dependent β has been found in the canonical P3HT:PCBM system with a recombination order of 2 (Maurano et al., 2010). Rare examples for β being carrier-independent (constant) are found in polymer solar cells based on PIPCP:PC_61_BM (Garcia-Belmonte et al., 2010). Generally, the recombination rate *B* follows,

(4) $$\begin{array}{l} B=\frac{dn}{dt_{carr}}=\beta np \end{array}$$

Through plotting *np* as a function of carrier lifetime *t*_carr_, the dependence of β on carrier density can be assessed. We extracted the carrier lifetime *t*_carr_ and carrier density *n* (here assuming *n* = *p*) by irradiation intensity dependent on impedance spectroscopy at open-circuit condition. In this situation, the net current flow is zero because of the compensation of charge recombination. The values of *n* were attained by integrating the measured chemical capacitance *C*_μ_ over *V*_oc_ at different *P*_light_, and *t*_carr_ was extracted from the product of recombination resistance and *C*_μ_ (Yan et al., 2018). Figure S4 shows Nyquist plots of impedance spectra measured on the solar cells at different *P*_light_. Through equivalent circuit modeling (see utilized circuit in Figure S5), the determined *n* is plotted against *P*_rad_ in Figure 5B. The *n* was found to fall in the range of 10^16^–10^17^ cm^−3^ at 1 sun (100 mW/cm^2^) irradiation, exhibiting a quasi-exponential dependence on *P*_light_. At 1 sun, the determined *n* in the binary solar cells roughly agrees with the value of ~5 × 10^−16^ cm^−3^ reported for other binary systems with similar band gaps (Yan et al., 2018). Consistently, the order of *n* follows the tendency of *J*_sc_ in device. It is noted that the *n* in the ternary device exhibits a sharper increase in the high light intensity regime (close to 1 sun), compared to the trend in binary devices. In a previous study, a saturation-like increase of *n* in DPP-containing SM-solar cells was found associated with more substantial carrier losses near 1 sun. (van der Poll Thomas et al., 2012) The trend in the ternary device in Figure 5B is suggestive of mitigated carrier losses near 1 sun irradiation. With the determined *n* and *t*_carr_, the recombination kinetics were assessed through plotting these two parameters in logarithm scale. As seen from Figure 5C, under the same carrier density, the ternary device is indeed associated with longer-lived photo-carriers. Considering that the *t*_carr_ is only governed by recombination in the open-circuit condition without involving transport, the longer lifetime of carriers should be mainly attributed to the reduced recombination rate with smaller values of β near approaching 1 sun, according to Equation 1. In a theoretical study based on an array of BHJ-OSCs, it was shown that the ratio of charge extraction time to recombination lifetime denoted as Θ can significantly affect FFs in solar cell (Bartesaghi et al., 2015; Ran et al., 2017). Typical Θ values in OSCs range between 10^−3^ and 10^−2^. Upon establishing an equilibrium between charge sweepout and recombination, increasing charge carrier mobility tends to shift the balance toward extraction. On this basis, a smaller value for the dimensionless parameter Θ can account for a higher FF. (Mozer et al., 2005) Based on the results of TPC and impedance spectroscopy, the Θ was determined to be 1.13 × 10^−1^ for the PCE-10:PC_71_BM binary device and 5.7 × 10^−2^ for the ternary device. Should be noted the Θ in operational solar cells may be even smaller as the charge extraction time determined by TPC was based on excitation with a laser pulse that has a much lower intensity than standard 1 sun irradiation. The reduced Θ in the ternary device reconciles the enhancement of FF, benefitting from the modified charge extraction and recombination balance.

The slopes in Figure 5C provide additional insights into the recombination order (RO). We found a slope of 2.14 in the BTR binary device, which resembles the value of P3HT:PCBM cells, pointing to roughly a 1st order recombination with RO roughly being 1. It suggests that the carrier density-dependence of β is weak in the SM-binary blends. The ternary device is associated with a slightly increased RO with a slope of 3.46, indicating a slight increase of the carrier dependence for β. Of interest, there appear two regimes with different slopes in the PCE-10 based binary cell. Variations of carrier density dependence or RO in different regimes of *P*_light_ have been found in previous (Maurano et al., 2010; van der Poll Thomas et al., 2012). A RO >1 is generally attributed to the effects of charge trapping/release, morphological traps, or spacial inhomogeneity in the distribution of photo-carriers (Andreas et al., 2011; Kirchartz and Nelson, 2012). These factors could play a role in our binary and ternary devices and further studies will be required to fully understand these observations.

With the identified impacts of recombination on the behaviors in ternary devices, at last we turn to the influence of BTR guest donor on *V*_oc_. For this purpose, we measured energy of charge transfer-states (CTS) with which the *V*_oc_ losses are assessable. CTS are ubiquitously present in OSCs and the energy of CTS (*E*_ct_, defined by the energetic distance from the ground states to the 1st excited states of CTS) sets an upper limit for experimentally achievable *V*_oc_ (Collins Samuel et al., 2015). As CTS are directly photo-excited, a commonly used method for determine *E*_ct_ involves measurements of the photocurrent arising from CTS absorption. Figure 5D shows Fourier transform photocurrent spectra (FTPS) of different solar cells. With the change of incident photon energy, we observe a wide range of photoresponse over orders of magnitudes. At the low energy regime, a sharp decrease of EQE is observed in association with an onset at 1.73 eV (PCE-10: PC_71_BM), 1.97 eV (BTR:PC_71_BM), and 1.72 eV (ternary). The difference in EQE onsets is consistent to that of the effective band gaps. The shoulder-like features are characteristics of the CTS absorption in fullerene based OSCs. The broad feature can be partially due to the relatively larger reorganization energy (λ) at CTS. (Perng et al., 1996; Vandewal et al., 2010) The values of *E*_ct_ and λ were determined through fittings of FTPS measurements to Marcus equation (see lines in Figure 5) (Marcus, 1990). As summarized in Table 2, the *E*_ct_ of ternary device is identical to that of PCE-10 binary device, being 1.44 eV. The *E*_ct_ of the SM-binary cell increases to 1.5 eV, related to the deeper-lying HOMO in BTR. We estimated the *V*_oc_ losses (Δ*V*_oc_) according to the relation, Δ*V*_oc_ = *E*_ct_-*V*_oc_ and the results are provided in Supplementary Material, SI. Among these BHJ solar cells, the least Δ*V*_oc_ (< 0.6 V) and the largest Δ*V*_oc_ (= 0.668 V) were found in the BTR and PCE-10 binary devices, respectively. It is of interest that the voltage losses become reduced in the ternary device, benefitting from the lower Δ*V*_oc_ in the BRT binary BHJ. This result points to the importance that the recombination losses for photo-carrier and voltage losses in the ternary solar cells can be simultaneously mitigated through the introducing the BTR guest with intrinsically a lower recombination and higher-lying *E*_ct_, eventually leading to the enhanced PCE. Based on the fitting results (see Table 2), the ternary device displays the smallest reorganization energy (λ = 0.231 V). The value of λ provides a measure of the line width or energetic disorder in CTS (Thomas et al., 2015). Generally, the reduction of λ benefits the delocalization of charge wave functions and charge separation (Graham et al., 2014). From the results of FTPS, the ternary device presents a reduced energetic disorder at the interfacial CTS. The morphology origin for this phenomenon may be linked to the higher domain purity in the ternary blends (Mario et al., 2011). The energetic offset between CTS and excited states in donor constitutes the radiative losses in *V*_oc_ (Shenkun et al., 2018). Meanwhile, it serves the critical driving force for interfacial charge dissociation. On this basis, for the sake of attaining smaller Δ*V*_oc_, it will be more realistic to suppress non-radiative losses in ternary devices without hampering charge generation and ultimate photocurrent. This may be implemented via control of transport balance and/or PL properties toward increases of electroluminescence efficiency in ternary BHJ films.

**Table 2:** Fitting parameters for FTPS measurements in Figure 5D according to the Marcus theory.

**BHJ**	***E*_**ct**_ (eV)**	**λ (eV)**	**Δ*V*_**oc**_ (V)**
PCE-10:BTR:PC_71_BM	1.44	0.231	0.646
PCE-10:PC_71_BM	1.44	0.256	0.668
BTR:PC_71_BM	1.50	0.339	0.596

## Conclusions

To summarize, we have comprehensively investigated the impacts of the introduced BTR small molecule guest to PCE-10:PC_71_BM binary blends on bimolecular recombination and photo-carrier losses in ternary solar cells. Strategically, we show that the reduction factor for recombination is modulated through co-blending the BTR donor with intrinsically a low recombination rate and balanced carrier mobility in blend with PC_71_BM acceptor. This leads to achieving a suppressed charge recombination and expedited charge sweepout in ternary devices. These modifications to some degree are correlated to charge transport characteristics in the ternary BHJ films, hinting that the mobility imbalance may not necessarily be a hindering factor. Based on the dimensionless parameter Θ that describes the ratio of charge extraction time to recombination time, the enhanced FF in the ternary solar cells is reconciled by the reduced Θ revealing the change of equilibrium between these two competing processes. By examination of recombination kinetics, we identify low recombination orders in the concerned BHJ systems, meaning a weak dependence of recombination rate constant on carrier density, possibly due to the specific nanomorphology in ternary blends. FTPS measurements indicate that the *V*_oc_ losses in the ternary cell are slightly mitigated upon addition of BTR, which originates from the rise of CT-state energy. The presented benefits in the presence of small molecule donor frame a useful guideline for future design of high efficiency ternary organic solar cells.

## Data Availability Statement

All datasets generated for this study are included in the article/Supplementary Material.

## Author Contributions

All authors listed have made a substantial, direct and intellectual contribution to the work, and approved it for publication.

### Conflict of Interest

The authors declare that the research was conducted in the absence of any commercial or financial relationships that could be construed as a potential conflict of interest.
