# Supplementary material for: Mediated Non-geminate Recombination in Ternary Organic Solar Cells Through a Liquid Crystal Guest Donor
Source: Front Chem. 2020 Feb 11;8:21. doi: 10.3389/fchem.2020.00021 (PMC7026665; doi:10.3389/fchem.2020.00021)
Supplement: Supplementary file 1 [file Table_1.DOCX]

**Supplementary Information**

Mediated Non-Geminate Recombination in Ternary Organic Solar Cells through a Liquid Crystal Guest Donor

Ao Yin^1^, Dongyang Zhang^1^, Jianqiu Wang^1^, Huiqiong Zhou^2,^*, Zhiqiang Fu^3^, Yuan Zhang^1,^*

^1^School of Chemistry, Beijing Advanced Innovation Center for Biomedical Engineering, Beihang University, Beijing, China

^2^CAS Key Laboratory of Nanosystem and Hierachical Fabrication CAS Center for Excellence in Nanoscience, National Center for Nanoscience and Technology, Beijing, China.

^3^School of Engineering and Technology, China University of Geosciences, Beijing, China

[yuanzhang@buaa.edu.cn](mailto:yuanzhang@buaa.edu.cn)


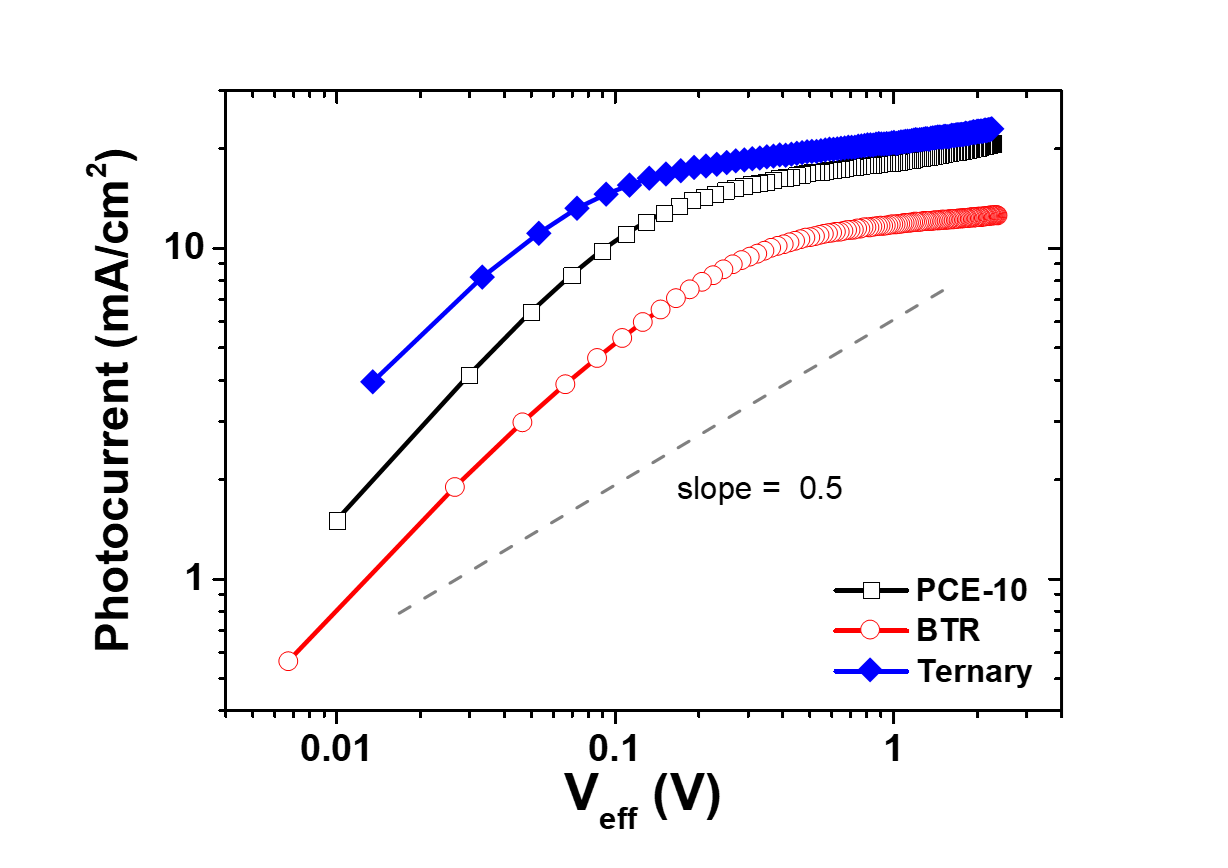


**Figure S1.** Photocurrent versus effective bias (V_eff_) characteristics of binary and ternary solar cells under 1 sun irradiation. V_eff_ is defined as V_eff_ = V_0_-V_app_ where V_0_ is the voltage corresponding to a zero photocurrent and V_app_ is the applied bias. Also compared by dashed grey line is the space-charge limited photocurrent (SCLC) with a square root dependence (slope = 0.5). Apparently, there is no observable SCLC in the concerned solar cells.


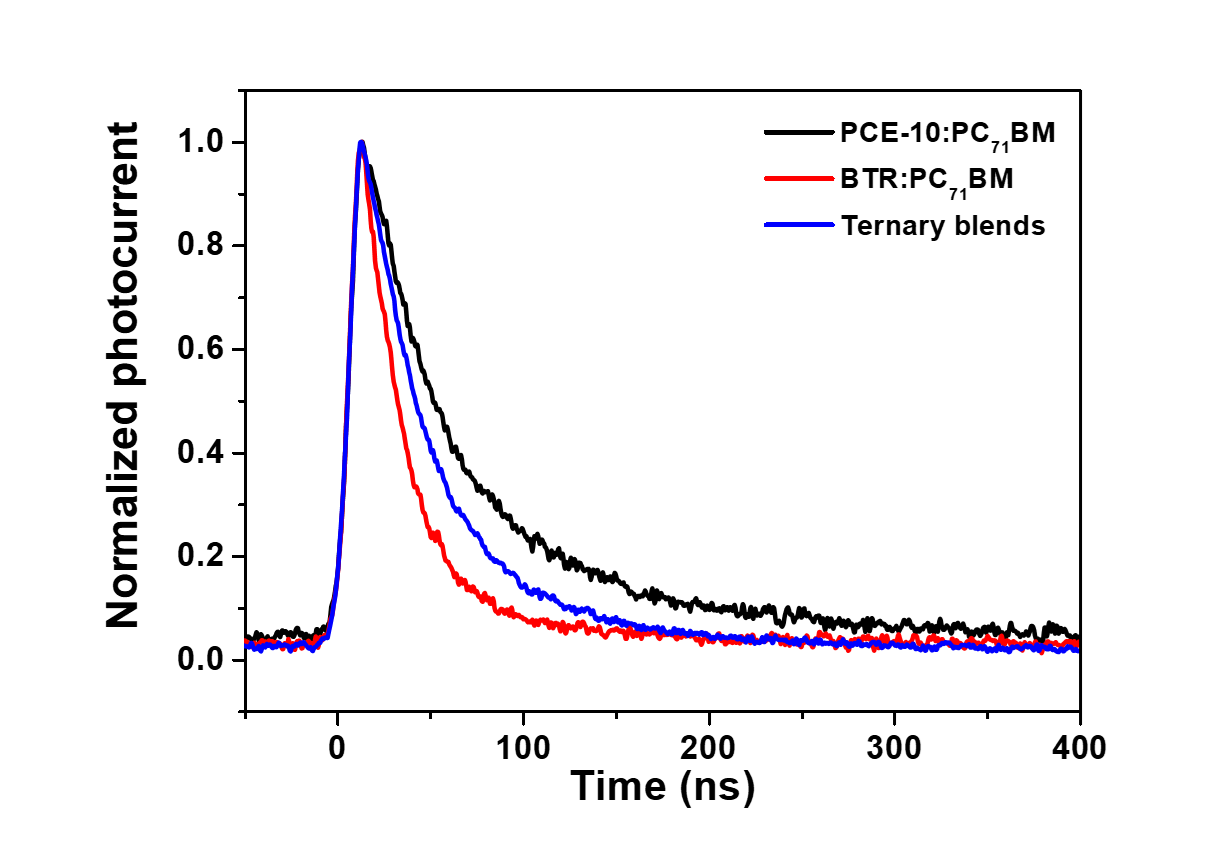


**Figure S2.** Normalized photocurrent decay kinetics of solar cells excited by a laser pulse at 488 nm without white light bias.

*
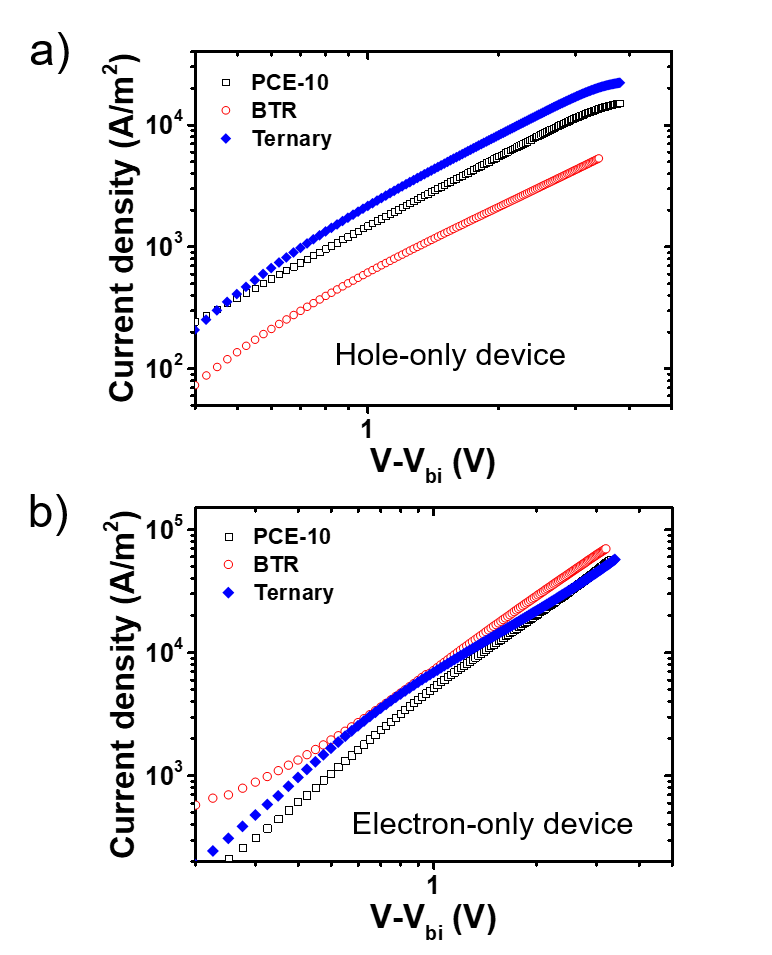
*

**Figure S3.** Dark current density versus voltage characteristics in double logarithms scale of (a) hole-only and (b) electron-only devices based on active layers comprising of various BHJ films. Obvious quadratic voltage dependence over a wide bias range is observed indicating a dominant space-charge limited transport behavior.

*
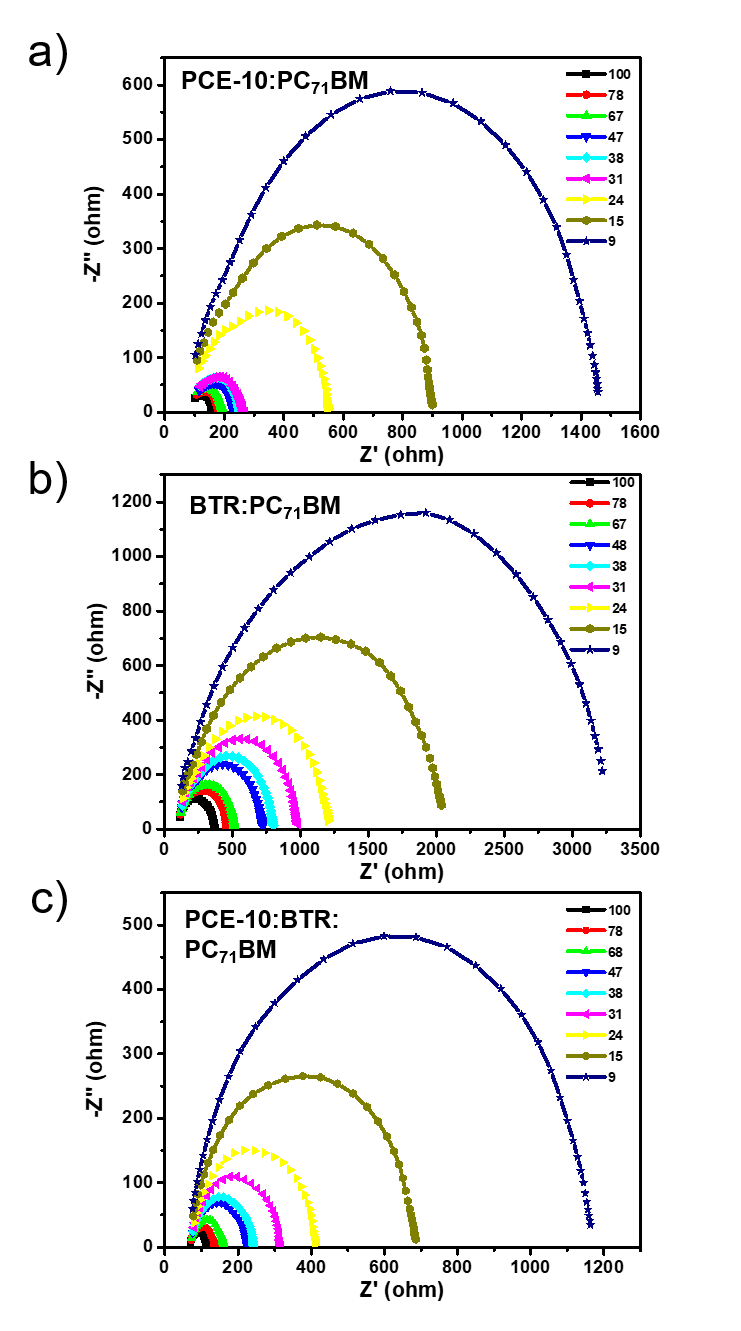
*

Figure S4. Irradiation-dependent Nyquist plot of impedance spectroscopy measured on various solar cells from which the carrier density and recombination lifetime are extracted.

*
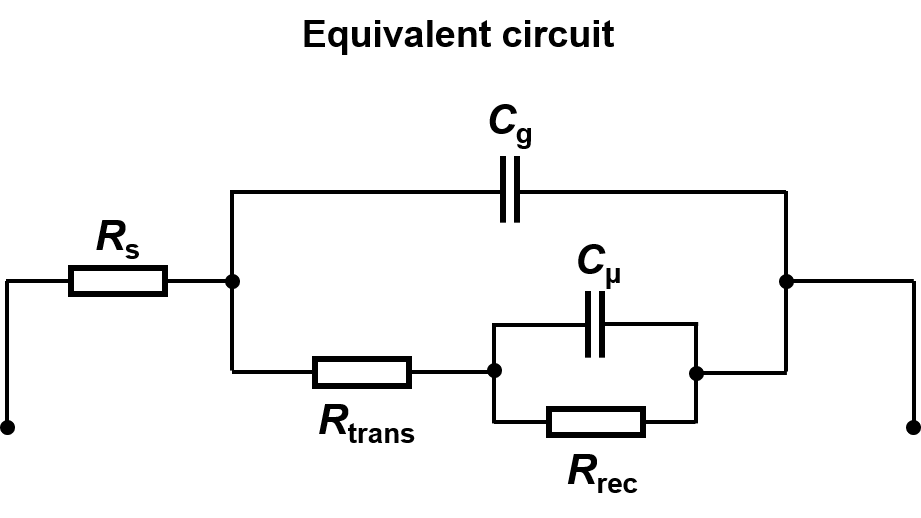
*

**Figure S5.** Equivalent circuit used to fit the Nyquist plots in Figure S4. R_s_, R_trans_ and R_rec_ is respectively the series, transport-related and recombination resistance. C_g_ and C_µ_ is the geometrical and chemical capacitance and the latter is the function of accumulated charges in the photoactive layer.
